# Longitudinal drug synergy assessment using convolutional neural network image-decoding of glioblastoma single-spheroid cultures

**DOI:** 10.1093/noajnl/vdad134

**Published:** 2023-11-05

**Authors:** Anna Giczewska, Krzysztof Pastuszak, Megan Houweling, Kulsoom U Abdul, Noa Faaij, Laurine Wedekind, David Noske, Thomas Wurdinger, Anna Supernat, Bart A Westerman

**Affiliations:** Laboratory of Translational Oncology, Intercollegiate Faculty of Biotechnology, University of Gdańsk and Medical University of Gdańsk, Gdańsk, Poland; Laboratory of Translational Oncology, Intercollegiate Faculty of Biotechnology, University of Gdańsk and Medical University of Gdańsk, Gdańsk, Poland; Center of Biostatistics and Bioinformatics, Medical University of Gdańsk, Gdańsk, Poland; Department of Algorithms and System Modeling, Gdansk University of Technology, Gdańsk, Poland; Department of Neurosurgery, Amsterdam UMC Location Vrije Universiteit Amsterdam, Amsterdam, The Netherlands; Cancer Center Amsterdam, Brain Tumor Center Amsterdam, Amsterdam UMC, Amsterdam, The Netherlands; The WINDOW Consortium (www.window-consortium.org); Department of Neurosurgery, Amsterdam UMC Location Vrije Universiteit Amsterdam, Amsterdam, The Netherlands; Cancer Center Amsterdam, Brain Tumor Center Amsterdam, Amsterdam UMC, Amsterdam, The Netherlands; The WINDOW Consortium (www.window-consortium.org); Department of Neurosurgery, Amsterdam UMC Location Vrije Universiteit Amsterdam, Amsterdam, The Netherlands; Cancer Center Amsterdam, Brain Tumor Center Amsterdam, Amsterdam UMC, Amsterdam, The Netherlands; Department of Neurosurgery, Amsterdam UMC Location Vrije Universiteit Amsterdam, Amsterdam, The Netherlands; Cancer Center Amsterdam, Brain Tumor Center Amsterdam, Amsterdam UMC, Amsterdam, The Netherlands; Department of Neurosurgery, Amsterdam UMC Location Vrije Universiteit Amsterdam, Amsterdam, The Netherlands; Cancer Center Amsterdam, Brain Tumor Center Amsterdam, Amsterdam UMC, Amsterdam, The Netherlands; Department of Neurosurgery, Amsterdam UMC Location Vrije Universiteit Amsterdam, Amsterdam, The Netherlands; Cancer Center Amsterdam, Brain Tumor Center Amsterdam, Amsterdam UMC, Amsterdam, The Netherlands; The WINDOW Consortium (www.window-consortium.org); Laboratory of Translational Oncology, Intercollegiate Faculty of Biotechnology, University of Gdańsk and Medical University of Gdańsk, Gdańsk, Poland; Center of Biostatistics and Bioinformatics, Medical University of Gdańsk, Gdańsk, Poland; Department of Neurosurgery, Amsterdam UMC Location Vrije Universiteit Amsterdam, Amsterdam, The Netherlands; Cancer Center Amsterdam, Brain Tumor Center Amsterdam, Amsterdam UMC, Amsterdam, The Netherlands; The WINDOW Consortium (www.window-consortium.org)

**Keywords:** convolutional networks, drug combination, glioblastoma, image processing, synergistic effect

## Abstract

**Background:**

In recent years, drug combinations have become increasingly popular to improve therapeutic outcomes in various diseases, including difficult to cure cancers such as the brain cancer glioblastoma. Assessing the interaction between drugs over time is critical for predicting drug combination effectiveness and minimizing the risk of therapy resistance. However, as viability readouts of drug combination experiments are commonly performed as an endpoint where cells are lysed, longitudinal drug-interaction monitoring is currently only possible through combined endpoint assays.

**Methods:**

We provide a method for massive parallel monitoring of drug interactions for 16 drug combinations in 3 glioblastoma models over a time frame of 18 days. In our assay, viabilities of single neurospheres are to be estimated based on image information taken at different time points. Neurosphere images taken on the final day (day 18) were matched to the respective viability measured by CellTiter-Glo 3D on the same day. This allowed to use of machine learning to decode image information to viability values on day 18 as well as for the earlier time points (on days 8, 11, and 15).

**Results:**

Our study shows that neurosphere images allow us to predict cell viability from extrapolated viabilities. This enables to assess of the drug interactions in a time window of 18 days. Our results show a clear and persistent synergistic interaction for several drug combinations over time.

**Conclusions:**

Our method facilitates longitudinal drug-interaction assessment, providing new insights into the temporal-dynamic effects of drug combinations in 3D neurospheres which can help to identify more effective therapies against glioblastoma.

**Key Points**

- Image processing based on convolutional neural networks (CNNs) allows us to estimate the viability in each well.
- Estimated cell viabilities can be used for synergy estimation at different time points.
- Our method allows for longitudinal assessment of drug interactions.

**Importance of the Study**

Understanding interactions between drugs over time is critical to estimate effectiveness and avoid therapy resistance. We show that convolutional neural network mediated image processing allows to estimate cancer cell viabilities that further enables the assessment of drug-interaction efficacy in a longitudinal fashion.

Glioblastoma (GBM) is the most common adult brain tumor and the median survival of patients is 14 months,^1–3^ where only 5% of the patients are alive after 5 years.^4^ Although GBM occurrence varies worldwide,^5^ in the United States, more than 12 000 glioblastoma cases are diagnosed each year.^6^ The last change of the standard of care to adjuvant Temozolomide was initiated in 1997,^7^ and follow-up efforts have shown only ineffectual success resulting in limited current options.^8^ Thus, there is still a need to discover new treatments for patients with GBM.

Drug combinations can increase a single drug’s effectivity or potency, minimizing toxicity and drug resistance, and are commonly used to treat cancer.^9^ GBM is a very heterogeneous tumor sensitive to drug combination treatment.^10,11^ There are 3 possible drug-interaction effects: additivity, synergy, and antagonism.^12^ Synergistic interactions are most desirable. To estimate drug interactions, 2 fundamentally different metrics Bliss Independence (BLISS^13^) and Loewe Additivity (LOEWE^14^) are commonly used. Another alternative is the Highest Single Agent (HSA)^15^ metric. Each metric captures distinct characteristics of drug interactions. Therefore, considering each of the mentioned synergy methods separately seems beneficial.^16^

The rapid advancement of image processing technology coupled with machine learning (ML) models allows for capturing biological interactions in an automated fashion. For this, an image (with cellular viability) must be converted into a mathematical object.^17^ The presence of noise, inadequate clarity, and poor contrast in images are recurring factors affecting image-based assessments.^18^ Trivializing, neural networks (NNs) are a series of ML algorithms that are inspired by the operations of a brain to identify relationships in data. The convolutional neural network (CNN or ConvNet) is a subtype of NNs commonly used in image and speech recognition.^19^ A specific type of CNNs that uses shortcut connections from shallow layers to deep layers is known as densely connected convolutional networks (DenseNet).^20^ DenseNet achieved state-of the-art performance in various image-processing tasks.^21^

The application of machine learning algorithms for the prediction of cell viabilities based on image processing was previously studied in cancer research through cytology,^22,23^ and in other research areas including musculoskeletal medicine,^24^ ophthalmology,^25^ and nephrology.^26^ Models^22,23^ were trained based on tissue images including many cells per image, whereas other^24–26^ focused on predicting viabilities not for cancer research. Furthermore, none of the published so far methods developed a cell viability predictive model based on single 3D neurospheres. Therefore, neither of these models could have been applied to our data.

In our study, we applied DenseNet architecture to build a predictive model for cell viability estimation. These results were further processed to provide synergy estimation. Consequently, the obtained synergies were used for the drug combination’s effect assessment.

## Materials and Methods

### Ethical Statement

All methods were carried out in accordance with relevant guidelines. Primary glioma sphere cultures were provided by MD Anderson Cancer Center, University Medical Center Groningen, and Massachusetts General Hospital in accordance with approval by the Institutional Review Boards. The use of tissues for experiments was exempt from requiring consent in all Institutes. Patient materials obtained from the University Medical Center Groningen were obtained after routine diagnostics, coded according to the National Code for the Good Use of Patient Material.

### Cell Culture, Image Acquisition, Viability, and Synergy Determination

Glioblastoma sphere cultures (GSC11 and GSC7-10) were obtained via single-patient surgical resections provided by Dr. Bhat (The University of Texas MD Anderson Cancer Center, Houston, TX, USA) and Prof. Sulman (NYU Langone’s Perlmutter Cancer Center/NYU Grossman School of Medicine, New York, NY, USA). Dr. Bakhos Tannous (Harvard/MGH, Boston, MA, USA) provided the GBM8 model. The chosen models are representative of different genetic subgroups of glioblastoma, that is, EGFR amplified (GSC11), PDGFRA amplified (GBM), or chromosome 7 gained (GSC7-10). All models were cultured in Neurobasal-A medium (NBM) supplemented with N-2 (100x), B-27 without vitamin A (50x), GlutaMAX™ supplement, 1% penicillin/streptomycin (all from ThermoFisher Scientific, Waltham, MA, USA), 20 ng/ml human EGF, 20 ng/ml human bFGF (Peprotech, London, UK), 5 IU/ml heparin (Amsterdam UMC pharmacy, Amsterdam, the Netherlands). Cell lines were cultured at 37 °C, 5% CO_2_. Single-cell suspensions were generated using Accutase (ThermoFisher Scientific, Waltham, MA, USA), by incubating neurospheres for 5 min at 37 °C, diluting with NBM, and resuspension of cell pellet in complete NBM. All GBM cultures were certified mycoplasma-free by regular testing via http://www.microbiome.nl/.

All tumors matched the IDH-wild type status, and diagnosis was based on histology analysis done by a neuropathologist. In case of lack of clarity of the tumor grade, an epic methylation profile was generated from paraffin-embedded tissue and matched to WHO criteria. Cell lines were also subjected to epic methylation array profiling allowing us to determine the copy-number profile, however, the interpretation of the subtype of tumor could deviate since there is no stromal infiltration in cell cultures as compared to clinical material.

Cell-repellent round bottom Cellstar® 96-well plates (Greiner, Alphen aan den Rijn, Netherlands) were used to seed cell cultures at a cell density of 750 cells/well allowing the formation of one spheroid per well for up to 18 days of culture. The protocol is based on earlier work.^27,28^ Three different patient-derived GBM cell lines, GBM8, GSC7-10, and GSC11, were selected for drug combination screening. These cell lines were selected intentionally since each of them exhibits different features that are prevailing for patients with GBM. GSC11 shows epidermal growth factor receptor (EGFR) amplification and GSC7-10 exhibits chromosome 7 gain but no obvious amplifications. GBM8 has shown EGFR diploidy and amplification in platelet-derived growth factor receptor A (PDGFRA) and MYCN instead.

Combenefit (“Combinations Benefit”) is complimentary and publicly available software that allows for estimating BLISS, LOEWE, and HSA synergistic and/or antagonistic drug combination effects assessment.^29^ It uses information about cell viability to make a proper drug-interaction estimation. Cell viability is defined as the number of healthy cells in a sample,^30^ and cell viability assays are used to measure the cell condition in response to drugs or chemical agents.^30–32^ Different types of assays are used to screen for outcomes in a process of most effective treatment development.^30^ The CellTiter-Glo 3D (CTG 3D) assay is a commonly used method to determine the number of viable cells in 3D cell culture. It uses ATP that is liberated from lysed cells which drives a chemiluminescent reaction that is measured by a luminometer. This approach claims to be more sensitive than other methods that depend on other metabolic mechanisms not always strongly activated in glioblastoma.^33^ CTG 3D-measured cell viabilities can then be used as an input for Combenefit.

Spheroid phase-contrast images were automatically taken with IDL virtual machine software via a Leica DMI3000 microscope (Leica, Rijswijk, the Netherlands) on a number of days throughout the experiment (1, 4, 8, 11, 15, and 18 days). On days 4 and 11, after images were taken, the Neurobasal-A medium (NBM) was revitalized by the replacement of 50 μl with fresh NBM. Secondly, drug combination treatments were applied on days 4 and 11 (drug combinations used in the study were in depth described^34^) for drug concentrations at ranges as described (Tables 1 and 2). Subsequently, on days 8 and 15, recovery periods were established throughout the removal of the drug combinations via complete refreshment of the NBM. Finally, on day 18, cell viability was measured by CTG 3D luminescent cell viability assay (Promega, Madison, WI, USA) according to the manufacturer’s protocol to assess the effect of drug combination treatment. Relative light units (RLUs) were measured via the Tecan Infinite® 200 reader using iControl 1.10 software, followed by normalization of the RLUs based on DMSO control (≤0.1% DMSO) to normalize it to the maximal cell viability (set to 100%). The data consist of technical duplicates of each of the 16 drug combinations.

**Table 1.** Drug Concentration Ranges for Drug Combination Screen.

Stock Concentration (mM)	Drug Range (μM)					
1	0.0500	0.0167	0.0056	0.0019	0.0006	0.0000
	0.1000	0.0333	0.0111	0.0037	0.0012	0.0000
10	1.0000	0.3333	0.1111	0.0370	0.0123	0.0000
	0.5000	0.1667	0.0556	0.0185	0.0062	0.0000
	2.0000	0.6667	0.2222	0.0741	0.0247	0.0000
	5.0000	1.6667	0.5556	0.1852	0.0617	0.0000

**Table 2.** Highest Used Drug Concentration Per Drug Per GBM Culture.

			GBM8		GSC11		GSC7-10	
			Maximum Concentration (μM) for 3-Fold Dilution					
#	Drug 1	Drug 2	Drug 1	Drug 2	Drug 1	Drug 2	Drug 1	Drug 2
1	CGP-082996	Obatoclax mesylate	5	0.50	5	0.50	5	0.50
2	Lapatinib	Gemcitabine	5	0.05	5	5.00	5	5.00
3	Lapatinib	Vinorelbine	5	0.05	5	0.05	5	0.05
4	Lapatinib	Obatoclax mesylate	5	0.50	5	0.50	5	0.50
5	Erlotinib	Gemcitabine	5	0.05	5	5.00	5	5.00
6	Erlotinib	Vinorelbine	5	0.05	5	0.05	5	0.05
7	CGP-082996	Thapsigargin	5	0.05	5	0.05	5	0.05
8	Lapatinib	Thapsigargin	5	0.05	5	0.05	5	0.05
9	Lapatinib	Tipifarnib	5	1.00	5	5.00	5	5.00
10	Lapatinib	Bleomycin	5	1.00	5	5.00	5	1.00
11	Tipifarnib	NVP-TAE684	1	0.50	5	0.50	5	0.50
12	Pazopanib	BMS-536924	0.1	1.00	5	5.00	5	1.00
13	PHA-665752	NVP-LAQ824	0.1	0.05	0.5	0.05	0.1	0.05
14	Thapsigargin	Midostaurin	0.05	0.05	0.05	1	0.05	1
15	Bleomycin	A-770041	1.00	1.00	5.00	1	1.00	5
16	Lapatinib	CGP-082996	5	5	5	5	5	5

Based on the data obtained from the experiment described above, the first objective of our study was to develop a cell viability prediction model that besides prediction on day 18 will also focus on viability prediction over time of the experiment (days 8, 11, and 15). That further allowed for longitudinal changes synergy estimation.

It is important to distinguish the difference between the 2 sets of collected data in our study. The first part consists of the data available on day 18, including pictures of cells and CTG 3D-measured cell viability. On day 18, photographs of all GBM cultures undergoing different drug combination treatments were collected. Drug combinations were tested in duplicates. Not including control cases, each plate contained 36 wells with cell cultures. One of 36 wells in each plate had no exposure to drugs. The remaining 35 wells were exposed to different doses of drugs. In an ideal scenario, with no manual image preselection, we would expect 3456 cell images per each day of our experiment. To most optimally train the prediction model, photos with poor quality or suspected staining were manually excluded, resulting in a total of 2728 photos collected on day 18. Photos taken on earlier days were not manually preselected, and all available images were used, regardless of the quality and noise, resulting in a set of 3456 pictures for each day. An additional set of 144 photos with empty wells was added to the train set as a reference.

Observed cell viabilities as determined by CTG 3D on day 18 served as labels for each cell photo taken on day 18 and were used in our predictive model. In the second data set (with data collected on days 8, 11, and 15) all available images were used regardless of the quality and the noise in the photos. Moreover, no CTG 3D cell viability equivalent was present for any of these images.

To develop a prediction model that is able to predict cell viability over time it was required to split all of our images into training and testing datasets. Photos for eight arbitrarily selected drug combinations were selected as a training set, and sensitivity analysis for a fully randomized selection of drug combinations as a train set was performed as well. The photos for the remaining eight drug combinations were used as an independent test set. Our model trained on the data available on day 18 and used all available photos on other days to make predictions, so there was no split into training and testing on days other than day 18. To address the concern of the differences in the size of cell cultures across different days, for each day we used the viability of cell culture with no treatment as the reference (100% viability) and normalized the remaining cell cultures on the given day accordingly. For both, the preprocessing and neural network, parameter tuning was performed using only the training set. Mean squared error was used as a measure for a model evaluation during the training process.

### Data Processing and Analysis

The 2 main goals in our study were to firstly accurately predict cell viabilities, and afterward use these predicted viabilities for synergy calculations. Prior to performing any prediction, image preprocessing was implemented.

**Figure 1.**
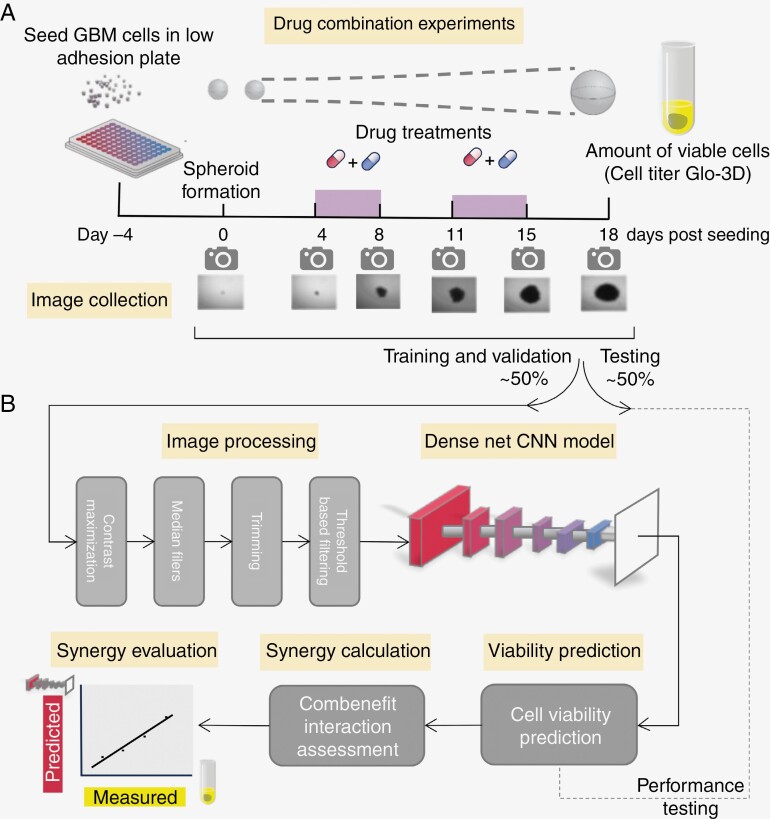
Biological experiment description and a pipeline of DenseNet-based CNN application to synergy prediction framework. A. Graph illustrating main study procedures. GBM cells were examined over 18 days time period. Images were taken on days 0, 4, 8, 11, 15, and 18. Drug combinations were applied between days 4 and 8, as well as days 11 and 15. Cell titer Glo-3D cell viability assessment was performed on day 18 only. Images under the axis with days show spheroid cell growth over time. B. Scheme of the DenseNet-based CNN framework used in the study. Images available on day 18 were divided into training and testing datasets. Automated preprocessing on all images was performed, and DenseNet-based CNN models were used for cell viability prediction. Observed through cell titer Glo-3D cell viabilities were used as labels in our prediction model. Combenefit software was used for further synergy estimation.

The following steps were taken in an image preprocessing (Figure 1B). First, contrast maximization was performed to minimize the impact of differences in lighting in the background. Subsequently, median filters and blurring were applied to reduce the number of remaining small artifacts. Since each image contained a large margin with no visible cells present, the margins were trimmed. The images were rescaled to 224 × 224 resolution. Additionally, threshold-based filtering was used to remove the remaining artifacts from the photographs.

Cell viability predictions were performed over time (days 8, 11, 15, and 18). Supervised DenseNet-based convolution neural network was adapted for the regression task. Predicted cell viabilities for the test set were compared with the readouts. For the readouts predicted and real viabilities, if data from both repetitions was available, the mean predicted and real viabilities were compared. If the data was available only for a single repetition, it was used instead of the mean.

The inferred cell viabilities were used as an input for the Combenefit drug synergy analysis. Estimated synergies from observed CTG 3D cell viabilities (real data) were available only on day 18. Synergies calculated from the predicted viabilities were compared with the synergies obtained from the real data.

### Missing Data

Combenefit uses matrices with cell viabilities to estimate the synergy of drug combinations, and missing values are not allowed in these matrices. Therefore, observed missing values in cell viabilities obtained from (CTG 3D) assay and predictive model were handled in the following manner: when any missing value in the matrix in the monotherapy well was observed, then that specific matrix was not considered for Combenefit analysis. At the same time, if the second matrix was complete, the calculation of synergy was based on this complete second matrix while the first matrix stayed excluded. Missing values in any other place than the monotherapy wells did not result in the exclusion of that specific matrix in a synergy calculation, the missing value was constituted with 100% viability.

In a model performance interpretation, any missing data in predicted or observed synergy or viability values for specific drug combinations resulted in the exclusion of the whole sample in the presented analysis.

## Results

### Data Preparation for Building Cell Viability Prediction Model

Longitudinal assessment of drug interactions enables to prioritize effective drug combinations with enduring effects. However, this assessment is currently challenged given that viability readouts are commonly determined as an endpoint. To perform a label-free assessment of longitudinal drug interactions, we set out to perform massive parallel monitoring of the cell viabilities for single neurospheres by using multiple recorded photographic images for each sphere. The key to such an effort is to be able to match extended sets of available images to individual neurosphere viabilities for different drug combinations and different GBM models. Available images would then allow us to estimate cell viabilities over time in a label-free fashion using image processing and machine learning.

We performed an experiment in a laboratory setting where 3 different GBM cultures were individually treated with 16 different drug combinations. Photographs were taken on several days, including the endpoint of day 18. We titrated 2 drugs in 3-fold dilutions from starting concentrations (see Tables 1 and 2). This dilution was performed in a 6 × 6 matrix where drugs are titrated individually but also diametrically in all combinations of 5 different concentrations using a solvent-only control as a reference for normalization. Experiments were performed in technical duplicates as 2 independent plates. The experiment description and data flow are shown in Figures 1A and B.

All images collected on day 18 were divided into 2 data set with an even split, 50% and 50% accordingly. Half of the samples were selected for training and validation purposes and another half for testing. Samples selected for training and validation were further split into training (70%) and validation (30%) sets. Both training and validation images were used for tuning the preprocessing step (some photos contained artifacts, the light was different etc.), but the training and validation split was preserved for model development purposes. Photos for 8 arbitrarily selected drug combinations (including Bleomycin and A-770041; CGP-082996 and Obatoclax Mesylate; Thapsigargin and Midostaurin; Lapatinib and Obatoclax Mesylate; CGP-082996 and Thapsigargin; Lapatinib and Thapsigargin; PHA-665572 and NVP-LAQ824; Lapatinib and Tipifarnib) were selected as a training set which contained 1437 images. The photos for the remaining 8 drug combinations were used as an independent test set, which consisted of 1291 photographs. Our model was trained and tested on the data available on day 18 only and therefore, no split into training and testing was necessary on other days than day 18.

### Image Processing Allows for Cell Viabilities Prediction

**Figure 2.**
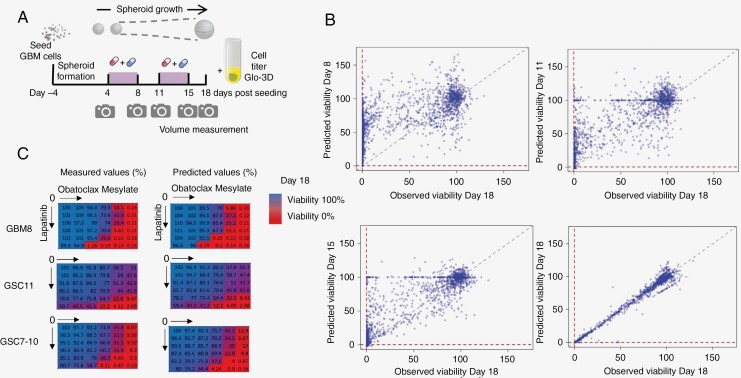
Accuracy of predicted cell viabilities. A. Illustration of the study design. B. Scatter plots of viabilities observed on day 18 versus predicted on other study days for all cell lines and all drug combinations (where both predicted and observed viabilities occurred) presented over the study period (days 8, 11, 15, and 18). Strong association on day 18 is presented due to the fact that the observed cell viabilities were obtained only on day 18. The remaining scatter plots relationships weaken over time, as expected. C. Exemplary observed and predicted viability heatmaps of dual drug combination of lapatinib and obatoclax in GBM8 (PDGFRA amplified), GSC11 (EGFR amplified), and GSC7-10 (EGFR gain) on day 18.

To assess drug interactions, the estimated cell viability effect of each drug or its combination should match the actual measured viability. To estimate this viability from the images, we applied contrast maximization, median filters, blurring, rescaled the images, and applied a Dense Convolutional Neural Network architecture. The measured viability based on CellTiter-Glo ATP measurements (CTG 3D) was taken to train the image-based model built on day 18 data. From this pre-trained model, viabilities were estimated at earlier time points, including days 8, 11, 15, and 18 (Figure 2A). Scatter plots of predicted on each day versus observed on day 18 viabilities are depicted in Figure 2B. As expected, a strong relation between observed and predicted viabilities can be seen on day 18 (adj *R*-squared = 0.984; *P*-value < 2.2e−16). This robust association was weaker when looking at earlier time points that indicate changes in predicted cell viabilities over time (day 15: adj *R*-squared = 0.730; *P*-value < .001 day 11: adj *R*-squared = 0.590; *P*-value < .001; day 8: adj *R*-squared = 0.429; *P*-value < .001). Additional model validation was performed and results can be found in Supplementary Figures S9–S10.

In addition, when experimental matrices with different drug combinations and concentrations were used, the results showed a remarkable match to measured values. That matching evaluation was reasonable to assess only on day 18, due to a lack of observed viabilities on other days of the experiment. Exemplary color-coded heatmaps for Lapatinib and Obatoclax Mesylate demonstrate a match between the predicted versus measured viabilities on day 18 stratified for the 3 different cell culture models (Figure 2C). Color in the heatmaps varies from blue to red, indicating the change in the viability, where red color shows low cell viability. Viability heatmaps on day 18 (and additionally on different days) for all drug combinations stratified by cell line are provided as Supplementary Figures S4–S8. These results indicate that it is possible to estimate the cell viability using machine learning on images, and in fact denote a proper setup to determine longitudinal synergy estimation.

### Predicted Viabilities Enable Longitudinal Synergy Assessment

**Figure 3.**
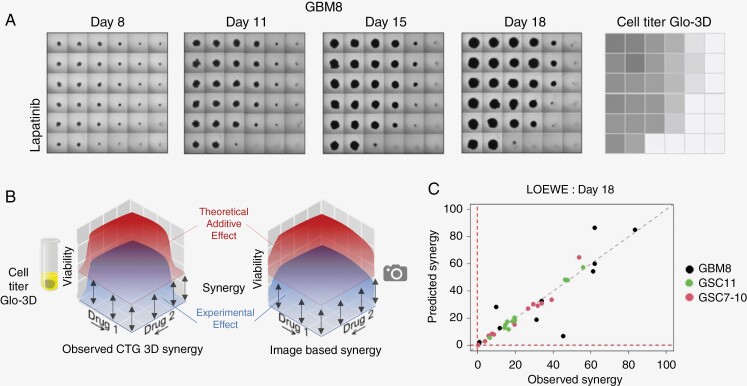
Synergy assessment based on predicted cell viabilities. A. Collected images of cell viabilities for lapatnib and obatoclax mesylate for GBM8 cell line, presented over time, along with the results from cell titer Glo-3D obtained on day 18. In the presented matrices, a top-left square always indicates the cells with no drugs applied. Bottom-right square indicates the cells with the maximal drug dose applied in the study. The first column and first row in each matrix represent the observed effect for monotherapies. B. Exemplary illustration of the complexity of the tasks performed by Combenefit for synergy estimation. Synergy is estimated by calculating the volume between the experimental (lower surface) and theoretical (upper surface) effect based on an additive interaction. In our study, synergy estimations were performed for all image-based predictions and cell titer Glo-3D obtained cell viability matrices. C. Scatter plot of LOEWE predicted versus observed synergy values for each cell line and each drug combination obtained on day 18 (where both predicted and observed synergies occurred) indicate (as expected) strong association.

**Figure 4.**
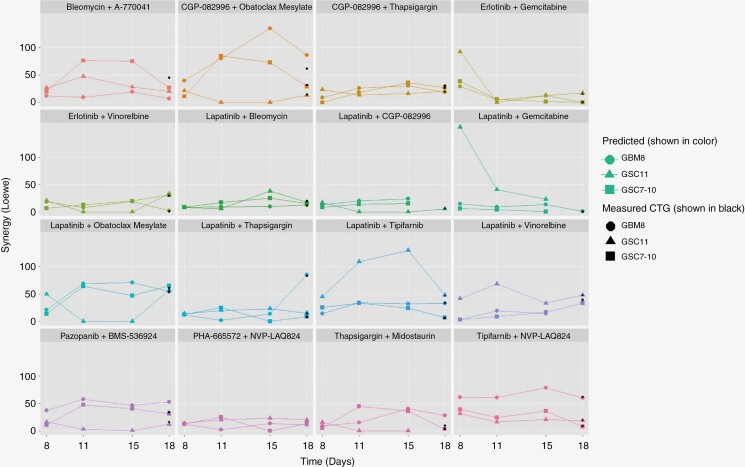
Longitudinal drug interaction assessment. Scatter plot of predicted (shown in colored symbols and lines) and observed LOEWE synergies (shown in black symbols at day 18) presented over study period stratified by cell line type and drug combination, showing mostly constant drug combinations effect over time.

Using the viabilities as determined by machine learning for the longitudinal data, we can estimate the level of drug interaction for each combination in time. In our study, we used Combenefit software to calculate the level of LOEWE, BLISS, and HSA drug interactions for each drug combination on a specific day (drug combinations applied only on days 4 and 11). Our interaction assessment is depicted in Figures 3A–3C, and 4. The first panel includes an example of how cell viabilities changed over time for the drug combination lapatinib and obatoclax mesylate in GBM8 to present how a synergistic drug effect would look like in the photos (Figure 3A). A summary of all photos used in the study can be found via data availability (github).

The concept of how to compare the calculated and measured levels of drug interactions is shown in Figure 3B. This figure underlines the complexity of the experiment since synergy is dependent on the expected effect of the curve-fitted monotherapies versus the effects of the drug combination. The downstream result of the viability predictions is ability to calculate synergies, and scatter plot with observed versus predicted LOEWE synergy on day 18 stratified by type of cell line is shown in Figure 3C. A strong correlation between observed and predicted synergies can be observed (adj *R*-squared = 0.837; *P*-value < .01). Results shown as Figure 3C are consistent across different synergy methods (see Supplementary Figure S1).

The level of estimated drug interaction calculated according to Loewe at day 18 is shown in Figure 4. A linear trend in the synergy values over time across different cell lines can be observed for a majority of considered drug combinations with a few exceptions. Erlotinib and gemcitabine as well as lapatinib and gemcitabine drug combinations for GSC11 show strong synergy at day 8 that decreases over time. Lapatinib and tipifarnib, along with CGP-082996 and obatoclax mesylate, present an increase in the predicted synergy values for GSC11 and GBM8, respectively, for both days 11 and 15, with a drop of the synergy at day 18. Lapatinib and obatoclax mesylate show a drop in predicted synergy values on days 11 and 15 for GSC11. All these nonlinear relationships are considered correct and were validated through manual image review to check for potential errors in the predicted model. Results shown in Figure 4 are consistent across different synergy methods (see Supplementary Figures S2 and S3). Observed CTG 3D synergies on day 18 were marked on the figures as black markers and show that predicted synergy values are consistent for the majority of drug combinations. Since observed CTG 3D synergies were obtained only at day 18, black markers are shown only at day 18.

Predicted cell viabilities in longitudinal setting allow us to estimate the synergy longitudinally. That results in a potential for more efficient drug combination assessment over time, which can directly improve the detecting most effective treatments for GBM patients. From the perspective of consistency among obtained predicted viabilities and synergy estimations, the following drug combinations were considered the most potent: A-77041 and bleomycin, CGP-082996 and obatoclax mesylate, and lapatinib and obatoclax mesylate.

## Discussion

GBM is a highly therapy-resistant tumor type, which might be caused by its intratumoral heterogeneity. Hence, drug combinations might affect heterogeneous cellular populations to increase therapy efficiency.^35^ For these drug combinations to be successful, they should provide a lasting effect to avoid therapy resistance. However, as most drug-interaction assessments are performed after 72 h of drug exposure, it is mostly unknown how drug interactions change over time beyond that time frame. Due to the powerful learning abilities of neural networks to recognize patterns, it becomes increasingly feasible to quantify biological phenotypes from image information, sometimes even minimizing human error.^36^ The growing increase in data availability and computing power has allowed CNNs to become commonly applied for image analysis.^36^ In our study, we proposed to use a CNN image-based machine learning model for a massive parallel longitudinal assessment of drug interactions in a label-free fashion.

We show here that GBM cell viabilities can be estimated using CNN image-based machine learning model in a consistent fashion over multiple laboratory cell culture models. Besides one-time point prediction, our model can be applied for longitudinal cell viability estimation. For numerous considered drug combinations (and especially for the ones with synergistic effect), changes in our predicted cell viabilities over time were found which translated to the level of synergy. For instance, some drug combinations have increased levels of synergy (eg, lapatinib and tipifarnib or CGP-082996 and obatoclax mesylate at days 11 and 15) and others decrease in time (eg, erlotinib or lapatinib in combination with gemcitabine).

Of the selected combinations identified in our study, a number have been identified before for different tumor types. CGP-082996 and obatoclax inhibiting CDK4 and BCL2, among other targets, have been shown to synergistically interact in mantle cell and follicular lymphoma.^37,38^ Furthermore MCL1 interacts with cyclin-dependent kinase 4 inhibitor C (P18INK4C), facilitating CDK4/6 mediated s-phase entry.^39^ Inhibiting EGFR and BCL family members similar to lapatinib and obatoclax, commonly shows positive interactions, also seen for glioblastoma, through different mechanisms including induction of endoplasmic reticulum stress, autophagy as well as increased MCL1 protein expression after EGFR inhibition.^40–43^ Application of erlotinib and gemcitabine has shown synergy in breast cancer models^44^ and in lung and pancreatic cells, synergistic interactions are mediated through nucleoside transport.^45^ However, for pancreatic cancer, this combination does not provide a significant improvement in patient survival.^46^ Lapatinib and gemcitabine application against breast cancer in a clinical setting provides a patient benefit^47^ where interactions might be depending on the scheduling. Lapatinib and tipifarnib both interact in the receptor tyrosine kinase and RAS/MEK/ERK pathway which could potentially lead to synergy through maximal pathway inhibition.^48^ Bleomycin A-770041 A-770041 acts through a cell-extrinsic mechanism in bleomycin-induced fibrosis.^49^

In view of the fact that drug effectiveness is understood to be different on day 8 of the experiment when the first drug combination doses stopped being distributed, versus at the end of the study period (day 18, after 2 rounds of the drug combination application). Predicted cell viabilities in our study were further used to assess the effectiveness of drug interactions over time, and obtained synergy curves were plotted showing a relatively progressively constant pattern (Figure 4). The predicted viability value for the reference data available on day 18 might have not only limited the viability assessment at earlier time points but might have affected the predicted level of drug interaction in our study. Therefore, drug interactions were calculated based on aggregated values for 36 wells thereby possibly averaging out individual viability estimation errors.

As we have collected numerous images, each matched to viability measurements, we could apply a machine learning algorithm for cell viability prediction in a longitudinal setting. Despite the large set of available images, the protocol for capturing photos was not fully standardized (different lighting conditions) and performed automatically by a microscope. That complicated the image preprocessing and potentially led to an underestimation of the model performance. Reduction of the 3-dimensional cultures to 2 dimensions (photos) is also expected to deteriorate the model’s ability to fully capture all the occurred changes in the cell size. While building a predictive model to estimate cell viabilities of GBM cells, we also accept that the lack of observed solvent-treated reference cell viabilities on days 8, 11, and 15, as well as the use of cell viabilities from day 18 as a reference, is a notable constraint. To compensate for the reduced spheroid sizes at these time points, the image window was adjusted such that it resembled the day 18 image. This normalization approach to handle differences in the size of cell cultures on different days and missing data in predicted viability matrices might have affected the performance of the model. Moreover, further research is needed to validate drug combinations effectiveness with in vivo experiments.

In summary, results from our study indicate that longitudinal drug interaction assessment utilizing automated imaging analysis is achievable. This non-invasive prediction, based on image data, has several advantages. It allows for continuous measurement of the approximate cell viability of the cultures during the experiment, without interfering with the later time points. The cost of taking additional measurements is low and primarily limited to the labor involved in preparing the additional set of images. It also provides the potential to investigate differences between various chromosomal alterations of GBM models, as well as recurrent GBM models. In conclusion, we have proposed a novel method for estimating cell viability to be used for synergy calculations for cancer cell lines. Overall, our method is intended to be shown as a proof of concept, adding to inspiring new insights into the discovery of synergistic drug combinations.

## Supplementary Material

vdad134_suppl_Supplementary_Figure_S1

vdad134_suppl_Supplementary_Figure_S2

vdad134_suppl_Supplementary_Figure_S3

vdad134_suppl_Supplementary_Figure_S4

vdad134_suppl_Supplementary_Figure_S5

vdad134_suppl_Supplementary_Figure_S6

vdad134_suppl_Supplementary_Figure_S7

vdad134_suppl_Supplementary_Figure_S8

vdad134_suppl_Supplementary_Figure_S9

vdad134_suppl_Supplementary_Figure_S10

vdad134_suppl_Supplementary_Data

## Data Availability

The data presented in this study are available on request from the corresponding author. Scripts, obtained viability heatmaps and summary of all used images can be find https://github.com/KrzysztofPastuszak/Longitudinal-drug-synergy-assessment. All analyses were conducted using R version 4.1.2 or higher, Python version 3.8 and Tensorflow version 2.7.
