# Supplementary figures and images for: Longitudinal drug synergy assessment using convolutional neural network image-decoding of glioblastoma single-spheroid cultures
Source: Neurooncol Adv. 2023 Nov 5;5(1):vdad134. doi: 10.1093/noajnl/vdad134 (PMC10691443; doi:10.1093/noajnl/vdad134)

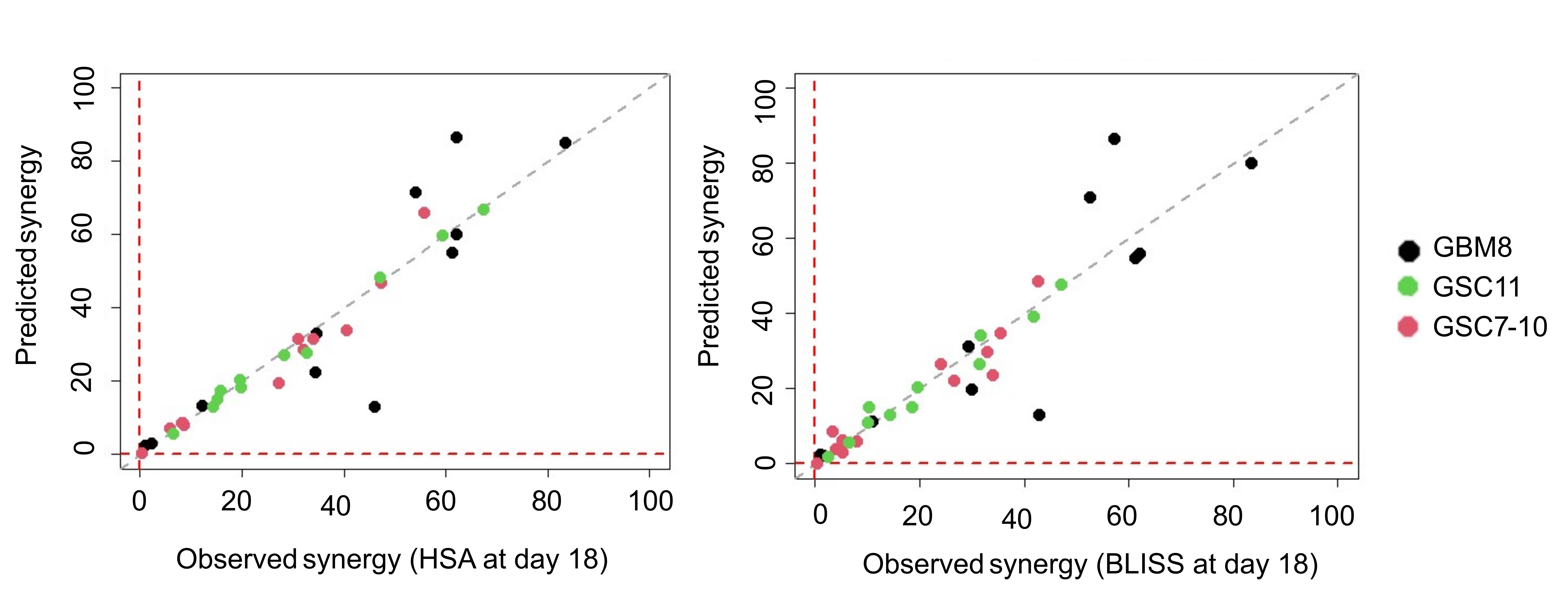

Supplement: vdad134_suppl_Supplementary_Figure_S1 [file vdad134_suppl_supplementary_figure_s1.jpeg]

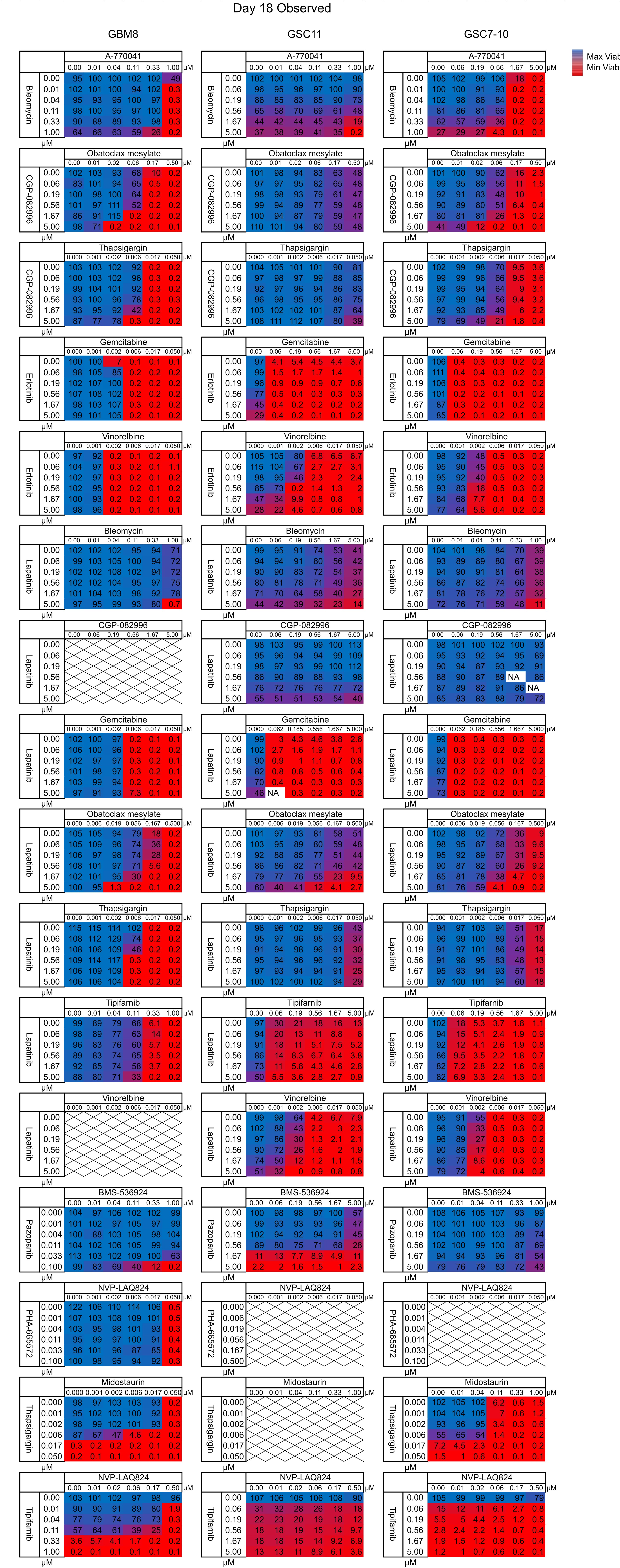

Supplement: vdad134_suppl_Supplementary_Figure_S4 [file vdad134_suppl_supplementary_figure_s4.jpeg]

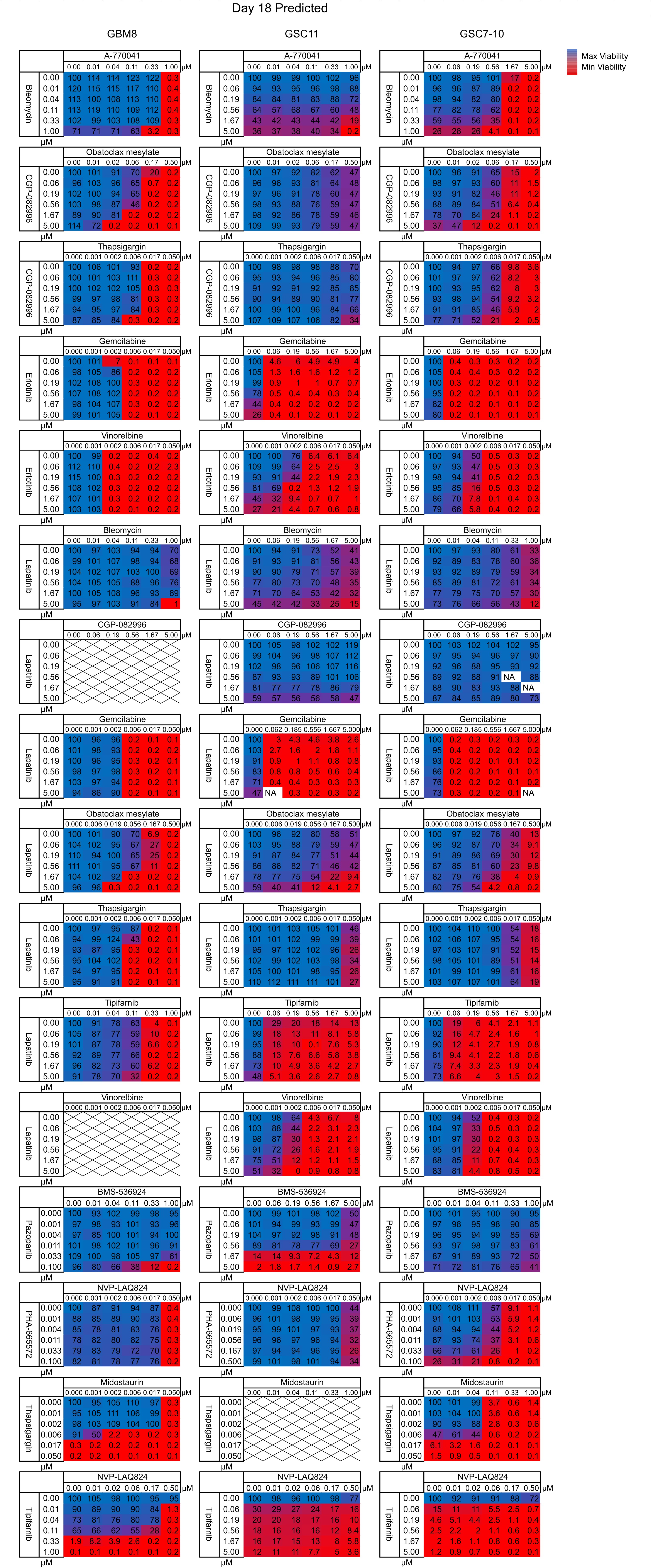

Supplement: vdad134_suppl_Supplementary_Figure_S5 [file vdad134_suppl_supplementary_figure_s5.jpeg]

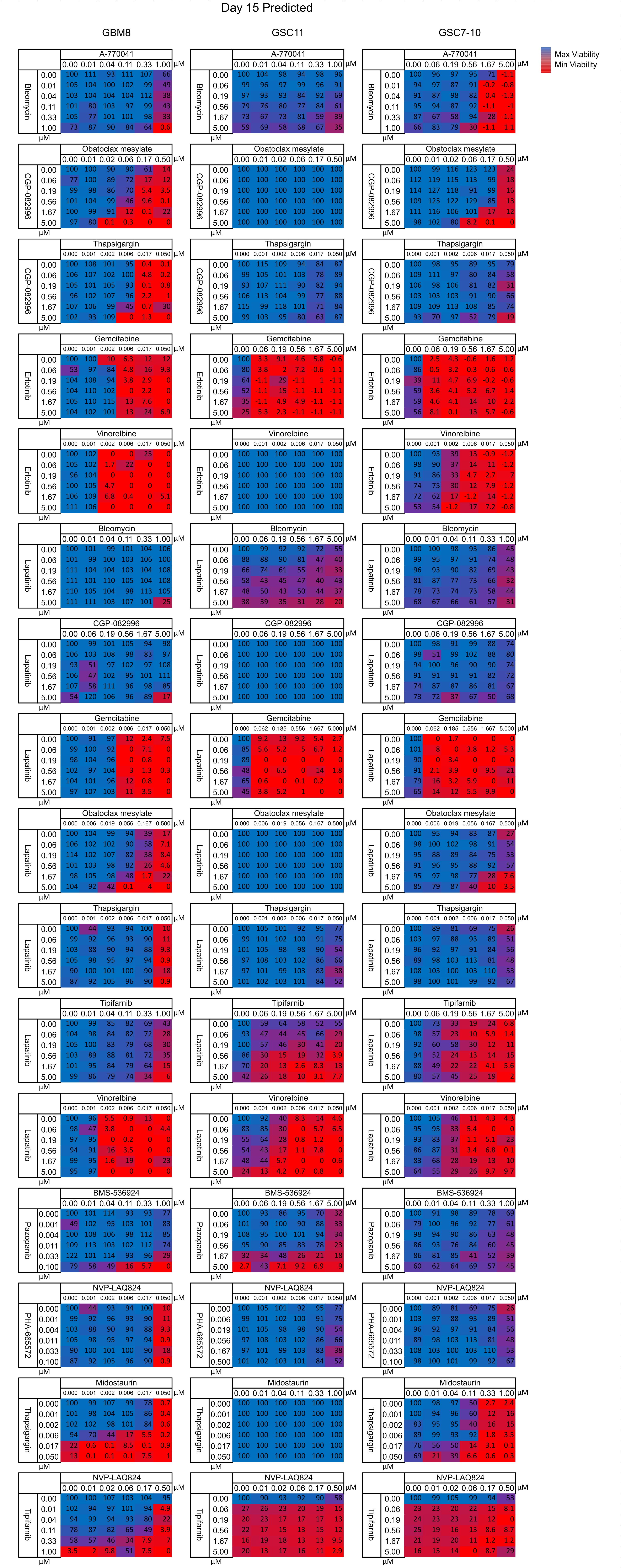

Supplement: vdad134_suppl_Supplementary_Figure_S6 [file vdad134_suppl_supplementary_figure_s6.jpeg]

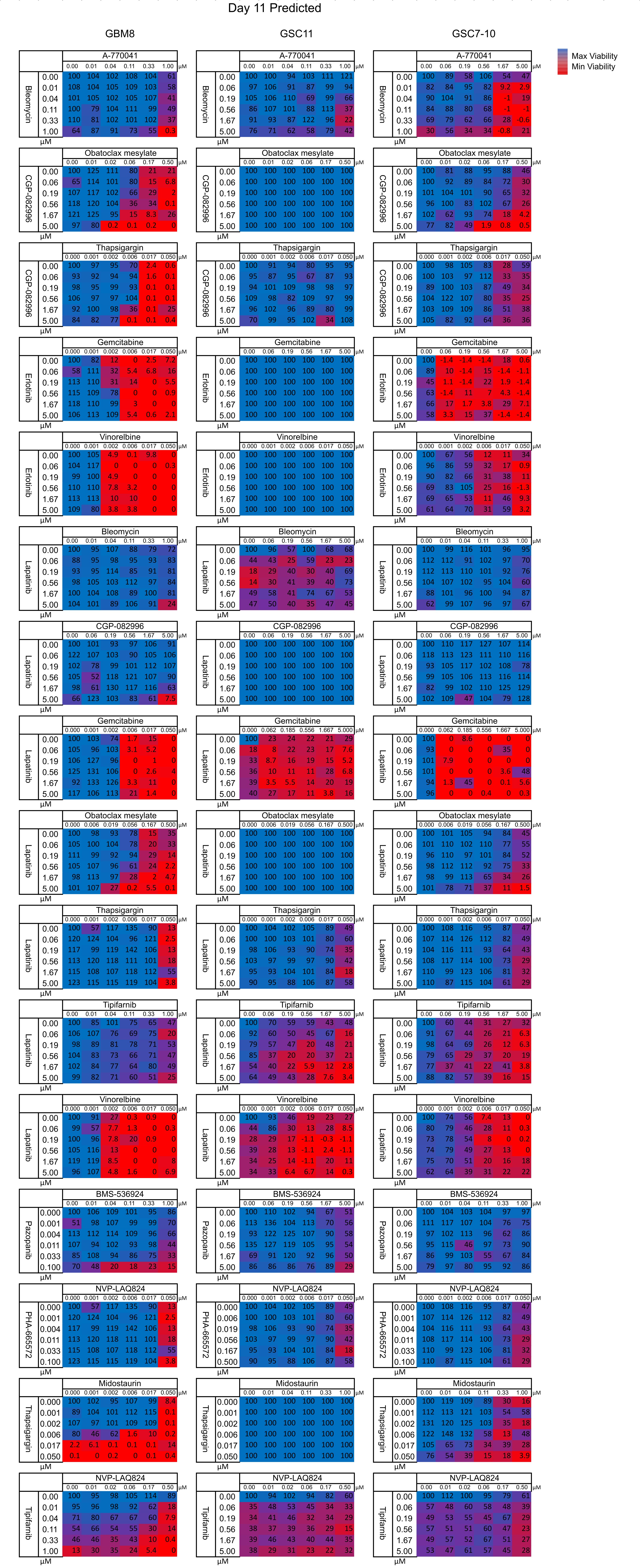

Supplement: vdad134_suppl_Supplementary_Figure_S7 [file vdad134_suppl_supplementary_figure_s7.jpeg]

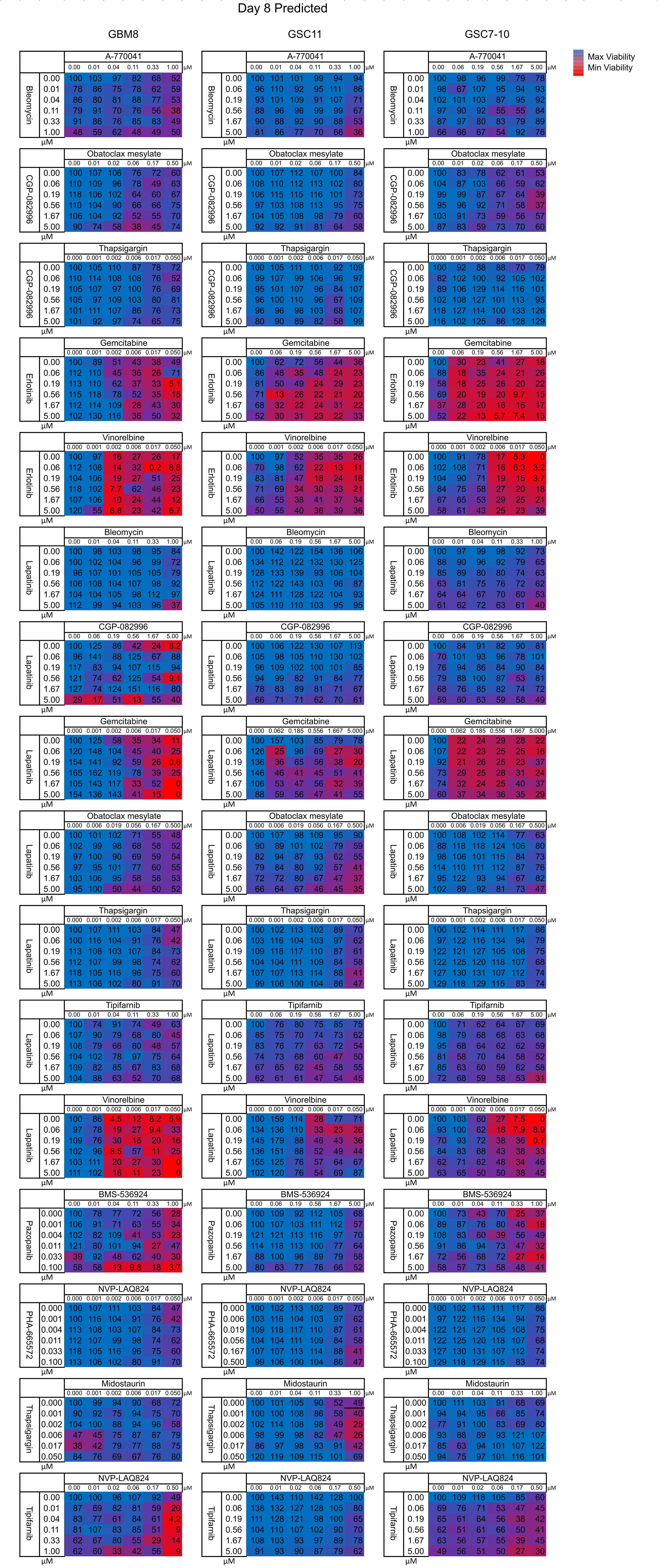

Supplement: vdad134_suppl_Supplementary_Figure_S8 [file vdad134_suppl_supplementary_figure_s8.jpeg]

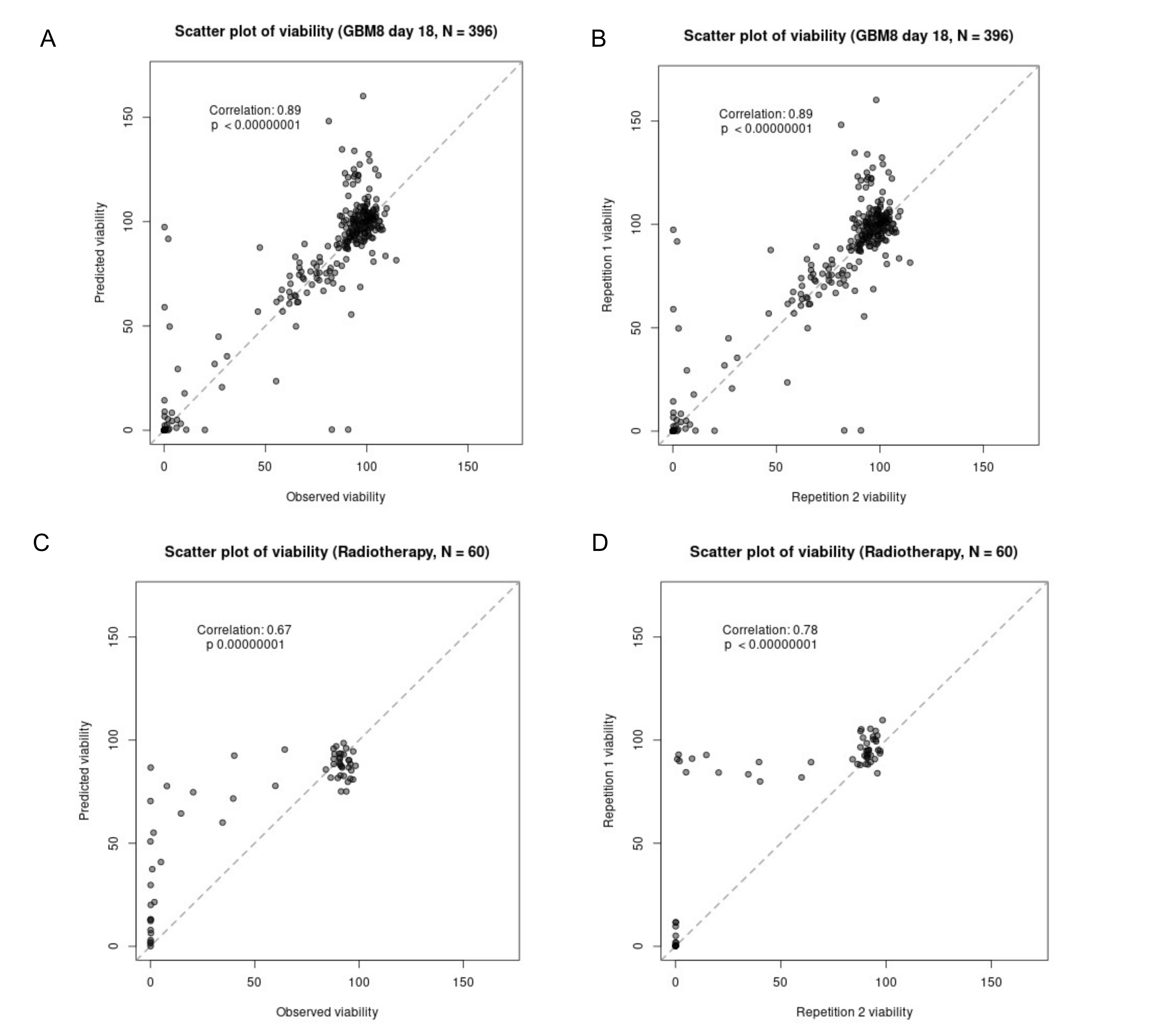

Supplement: vdad134_suppl_Supplementary_Figure_S9 [file vdad134_suppl_supplementary_figure_s9.jpeg]

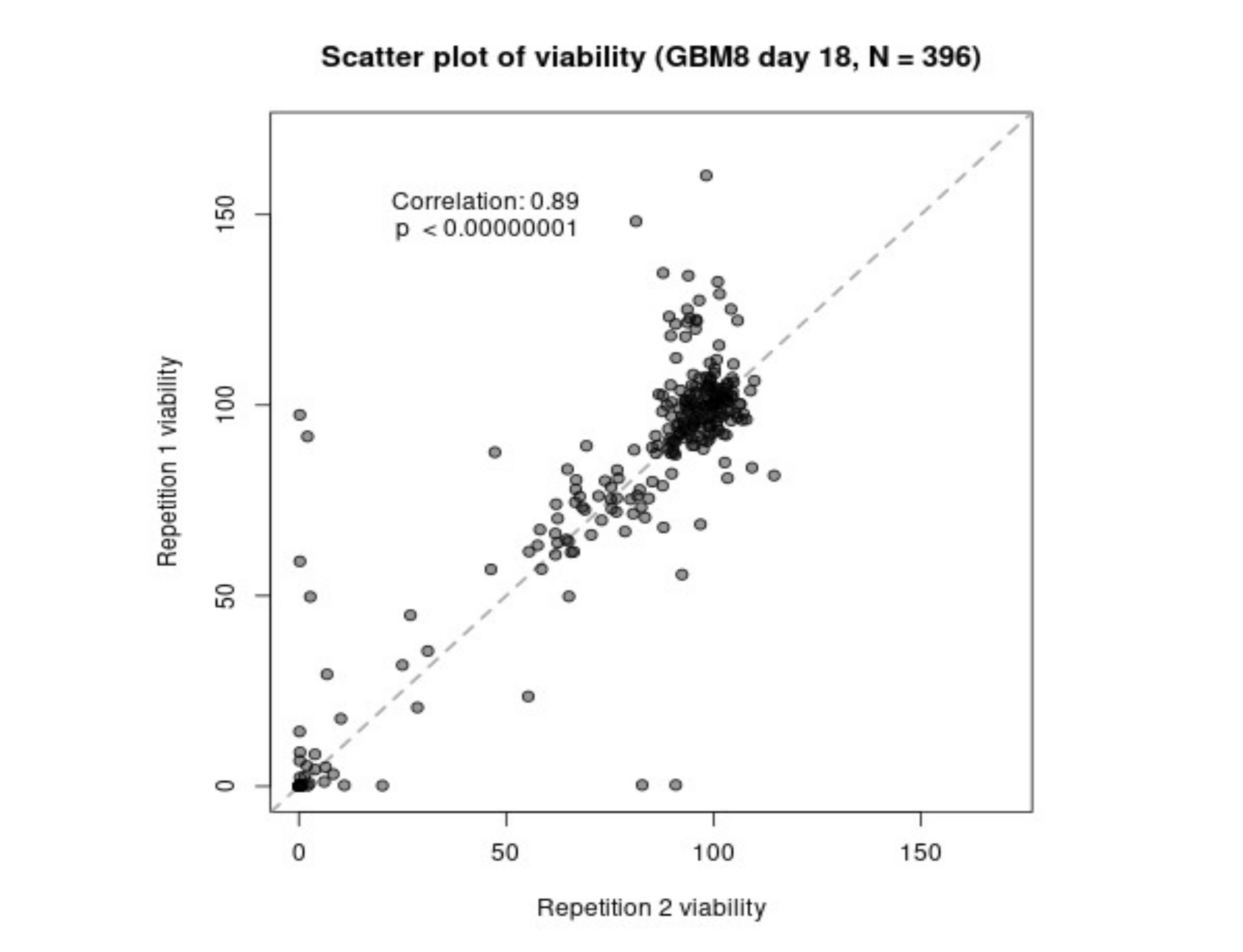

Supplement: vdad134_suppl_Supplementary_Figure_S10 [file vdad134_suppl_supplementary_figure_s10.jpeg]
