# Supplementary material for: Longitudinal drug synergy assessment using convolutional neural network image-decoding of glioblastoma single-spheroid cultures
Source: Neurooncol Adv. 2023 Nov 5;5(1):vdad134. doi: 10.1093/noajnl/vdad134 (PMC10691443; doi:10.1093/noajnl/vdad134)
Supplement: vdad134_suppl_Supplementary_Data [file vdad134_suppl_supplementary_data.docx]

**Supplemental materials**

**Figure S1.** Scatter plot of HSA and BLISS predicted vs observed synergy values for each cell line and each drug combination obtained on day 18 (where both predicted and observed synergies occurred) indicate (as expected) strong association.

**Figure S2. Longitudinal drug interaction assessment.** Scatter plot of predicted and observed HSA synergies presented over study period stratified by cell line type and drug combination.

**Figure S3. Longitudinal drug interaction assessment.** Scatter plot of predicted and observed BLISS synergies presented over study period stratified by cell line type and drug combination.

**Figure S4. Heatmaps of observed viabilities on day 18.**

**Figure S5. Heatmaps of predicted viabilities on day 18.**

**Figure S6. Heatmaps of predicted viabilities on day 15.**

**Figure S7. Heatmaps of predicted viabilities on day 11.**

**Figure S8. Heatmaps of predicted viabilities on day 8.**

**Figure S9. Additional model validation.** We have performed additional experiment where cell lines GSC7-10 and GSC11 were selected for training and validation purposes. 30% of the samples were used as the validation set and remaining 70% as the training set. GBM8 samples were used as the independent test set. Samples that had only one repetition available were omitted. **Panel A**: represents correlation between predicted and real viabilities. Further, we measured the correlation between biological replicates (each drug combination was included in the experiments in two repetitions) to evaluate the performance of the model. **Panel B**: shows correlation between real viabilities from first and second repetitions. Some difference in distribution were observed, but general level of correspondence was almost the same. Since the viability studies are often conducted to analyze drug efficacy, the final synergy analysis is less prone to the single errors, as long as the general trend is similar. Furthermore, we gathered data from another set of experiments, where GBM8 cell cultures were subjected to radiotherapy treatment. **Panel C**: shows results for one of the repetitions for radiotherapy experiment. **Panel D**: presents difference between real values for both repetitions in radiotherapy experiment. There is a bigger disparity between predicted and real values than between real values from two repetitions, however this level of differences should not significantly affect the results of further synergy analysis.

**Figure S10. Additional model validation.** For each drug and cell line combination, we had two repetitions. Further validation of results stability between repetitions was made and correlation of actual viabilities of the readouts between two repetitions for one of the cell lines are presented on the figure. Despite observed strong correlation, some level of variability between repetitions is noticeable.
